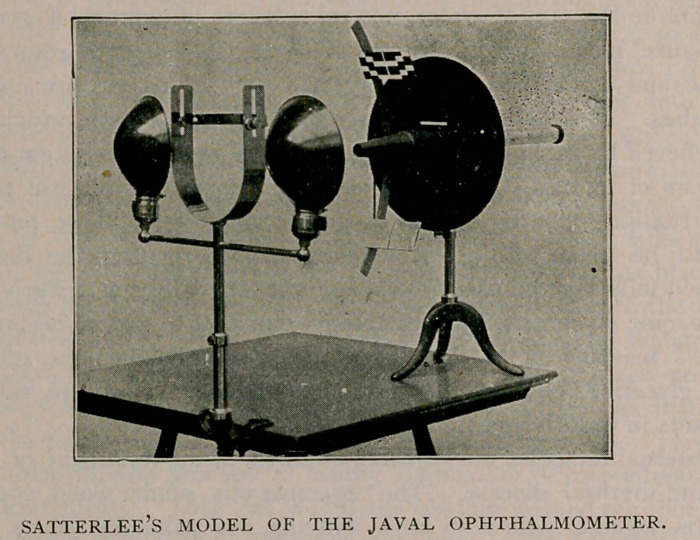# The Javal Ophthalmometer—Satterlee’s Model

**Published:** 1898-05

**Authors:** Richard H. Satterlee

**Affiliations:** Buffalo, N. Y.


### NEW INSTRUMENT.

### THE JAVAL OPHTHALMOMETER—SATTERLEE'S MODEL.

By RICHARD H. SATTERLEE, M.D., Buffalo, N. Y.

NINETY-EIGHT per cent. of astigmatism is corneal. Astigmatism is a frequent cause of reflex disturbances. The ophthalmometer is the only instrument that will determine the existence or absence of astigmatism without depending entirely upon the intelligence of the patient.

The objection to the Javal-Schiötz ophthalmometer, or its American copy, has been its cost, its size, and the number of lens surfaces, making very bright illumination of the mires necessary.

SATTERLEE'S MODEL OF THE JAVAL OPHTHALMOMETER.

In the model illustrated the number of lens surfaces have been decreased, making it possible to use the instrument by daylight, and lessening the cost of the instrument.

The Javal, as ordinarily constructed, is too unwieldy to transport. This instrument has been constructed so that it can be taken apart and will go in an ordinary grip.

The instrument is made for the Buffalo Ophthalmometer Co. of this city. Address P. O. Box 34, Buffalo, New York.

## Figures and Tables

**Figure f1:**